# Adapting lessons from the Indian subcontinent to accelerate elimination of visceral leishmaniasis as a public health problem in East Africa

**DOI:** 10.1371/journal.pntd.0014088

**Published:** 2026-03-13

**Authors:** Eva Iniguez, Daniel Masiga, Caryn Bern, Sridhar Srikantiah

**Affiliations:** 1 Vector Molecular Biology Section, Laboratory of Malaria and Vector Research, National Institute of Allergy and Infectious Diseases, National Institutes of Health, Rockville, Maryland, United States of America; 2 International Centre of Insect Physiology and Ecology (icipe), Nairobi, Kenya; 3 Department of Epidemiology and Biostatistics, University of California San Francisco, San Francisco, California, United States of America; 4 Bihar Technical Support Program, Patna, Bihar, India; Advanced Centre for Chronic and Rare Diseases, INDIA

## Abstract

This viewpoint draws lessons from the elimination of visceral leishmaniasis (VL) as a public health problem in the Southeast Asia region (SEAR) to inform efforts in East Africa (EA), now the global epicenter of *Leishmania donovani* transmission. VL is fatal and there is no licensed vaccine. Success in India relied on robust surveillance, rapid diagnosis, single-dose treatment, vector control, and multi-partner coordination. EA faces additional challenges than SEAR with multiple sand fly vectors, sensitive diagnostics and longer treatment regimens, high population mobility, and gaps in ecological and epidemiological knowledge. We highlight how strategies from South Asia could be adapted while acknowledging EA’s unique ecological and health system complexities. These insights aim to guide sustainable VL control towards elimination of VL as a public health concern in the region.

## Recommendations for East African VL elimination programs

The success of programs to eliminate visceral leishmaniasis (VL) as a public health problem in the Southeast Asia region (SEAR) has generated optimism that similar success can be achieved in East Africa (EA), the current epicenter of *Leishmania donovani* transmission [1]. In SEAR, humans with VL or post-kala-azar dermal leishmaniasis (PKDL) constitute the proven infection reservoir; the VL case load fell by >95% after implementation of active case detection (ACD), rapid diagnosis and single-dose liposomal amphotericin treatment. Extensive indoor residual spraying was conducted, but its contribution to VL elimination remains contested.

Control in EA is more challenging than in SEAR [1,2]. Infected animals have been repeatedly documented [3,4], though the zoonotic contribution to the human VL disease burden remains unclear. Transmission occurs via multiple sand fly species with differing bionomics and distribution, while evidence-based vector-control strategies are lacking [1]. A concerted effort will be needed to enable effective surveillance and to confront epidemiological complexities. This viewpoint reviews lessons from SEAR that may help to inform program design and implementation in EA (Box 1).

In SEAR, the elimination target was defined as subdistrict incidence <1 VL case per 10,000 population. No epidemiological or biological evidence exists to predict long-term sustainability based on a specific incidence target. In EA, the proposed targets are more complex, including regional VL incidence, time to treatment post-diagnosis, child deaths, HIV-VL treatment coverage, and PKDL incidence [1]. Objective assessment will require reliable surveillance for VL, HIV-VL, and PKDL plus accurate tracking of treatment and deaths.

In India, reliable village-level incidence estimates were established about a decade before the elimination threshold was achieved, providing high confidence in case counts [5]. SEAR surveillance systems evolved from earlier systems, refined based on program needs, health system structure and epidemiologic patterns observed over time. The design ensured close to universal capture and tracking, even for cases treated outside their residence region [5]. It relied on multiple data sources and process indicators, enabling triangulated understanding of successes and failures in real time. ACD options were evaluated [6], and vector surveillance was led by both governmental and non-governmental partners [7].

As in SEAR, encouraging multiple surveillance approaches will be valuable in EA. Establishing practical indicators for surveillance quality at the outset is crucial, anticipating diminishing system sensitivity as incidence falls and attention wanes. In EA, the effort and creativity demanded will likely be greater than in SEAR to achieve similar confidence; low population density, migratory populations, and cross-border movements demand different designs for baseline assessments and ongoing tracking.

Despite India’s thriving private healthcare sector, patients eventually sought care in the public sector because of lower costs and evident effectiveness, especially once rapid diagnosis and improved treatment regimens were implemented in peripheral health care facilities. Similarly, a strong network of effective subsidized services in EA should largely suffice to attract patients and reduce the need for intensive behavior change communication efforts. Local informants were useful complements to the formal system of village health workers, an approach EA programs may benefit from.

In India, fever was not always the first reported symptom of VL; some patients reported only prolonged malaise or anorexia. The “suspect case” definition should be tailored to local idioms and flexible enough to encompass variability. Implementation research will be crucial: could healthcare volunteer networks help identify and track suspects? Are there ways to decentralize diagnosis and treatment protocols to balance technical rigor and field-friendly approaches?

An idea that kept cropping up in SEAR, though never implemented, was to integrate VL into a fever management algorithm, including rapid diagnostic tests for malaria and other febrile diseases. This is likely even more relevant in EA and could contribute to a more ‘horizontal’ program from the outset, rather than pursuing elimination through vertical programs and later struggling to integrate them into existing systems.

Management systems must encompass patients from across porous provincial and international borders, including countries (e.g., Chad, northern Cameroon) outside classic foci where human cases may go undetected. WHO can help ensure a liberal public health outlook, mitigate suffering, assuring care, increasing trust in health systems, discover hitherto unknown transmission foci, and recover otherwise lost data.

The relatively low sensitivity of rK39 rapid tests in EA increases misdiagnosis risk and necessity for invasive sampling and/or prolonged follow-up [8]. Unlike the single-dose regimen in SEAR, effective treatment in EA requires ≥2 weeks of daily injections of two drugs [1]. Combined with difficult access to treatment centers due to long distances, this impedes full compliance and raises the specter of acquired resistance. Treatment completion among VL-HIV co-infected patients is crucial, as these individuals may act as superspreaders [4]. HIV prevalence in EA is higher than in SEAR, and diagnosis by rK39 is much less sensitive in VL-HIV. One idea mooted in SEAR, but never implemented, was providing insecticide-treated nets to VL/PKDL/HIV suspects to prevent transmission. In EA, a few nets used by at-risk communities or migrants with early symptoms might decrease transmission at a low cost.

Appropriate, evidence-based vector control strategies are needed for EA. In SEAR, IRS was instituted without research demonstrating effectiveness to prevent VL, based on the assumption that *Phlebotomus argentipes* was strongly endophagic and endophilic. High coverage was achieved, but there is little direct evidence that IRS contributed substantially to incidence reduction [7]. This may reflect recent findings showing a high proportion of *Ph. argentipes*, including infected flies, captured in vegetation [7,9]. African programs should be more frugal and rational in their strategies, as the predominant vector species are exophilic and exophagic [10]. Effective vector control in EA will demand innovative strategies, such as outdoor interventions tailored to the ecology and human behavior of specific localities, and should be based on pragmatic trials and coordination.

One important factor in SEAR success was oversight by district-level bureaucrats with substantial authority. In EA, subnational oversight systems may not be as well developed. VL elimination strategies will need to overcome limitations of existing health systems, or, better still, strengthen system ‘horizontals’, demanding broad public health capacities, not merely VL expertise.

Population densities in EA are generally low, often less than 10/sq km, versus affected SEAR rural areas of ~1000/sq km. Program logistics must adapt to these realities. For instance, redundancies and consequent ‘wastage’ of diagnostics and therapeutics should be anticipated and budgeted for; creative transport mechanisms will be needed. Again, these challenges demand expertise beyond the conventional. Even at SEAR densities, systems struggled to maintain material availability, particularly as incidence fell. The contrasting association of VL incidence with high population densities in SEAR vs persistence in low population densities in EA may also hold clues to transmission dynamics, for example, the role of non-human infection reservoirs.

Perhaps the most important, though generally overlooked, reason for SEAR success was the effective decentralization of governance of process integrity, such as tracking cases from suspicion to complete treatment, monitoring stock availability, and overseeing IRS operations. This was driven by centralized and decentralized efforts, by system actors and partner teams. Something similar will be needed in Africa.

Involvement of multiple partners supporting government efforts was crucial in SEAR, serving multiple purposes—bringing in different approaches, providing additional resources for oversight, contributing to early identification of successes and lapses in operations, redesigning microsystems, etc. Differing perspectives sometimes led to acrimony, but all partners contributed to success. Partner efforts may be even more crucial in EA considering the immense contextual variation in disease patterns and health systems. Uniting partners toward the ultimate goal is critical, but attempting to unite them at operational levels is likely to be self-defeating. Open, empathetic, evidence-based dialogue and mutual learning can form the basis of partnerships.

The complementary roles played by SEA partners were possible because flexible funding was available for aspects where the government system was not nimble enough. For instance, partners were able to quickly mobilize resources for field oversight, including transport, development of various subsystems and trainings, as well as for interim commodities procurement. Such “technical assistance” will be invaluable and synergistic in EA. Efforts of decentralized health system actors will be facilitated by flexible financing from World Bank or a similar funder, recognizing that VL is not just a public health burden, but also one that affects productivity and economic development.

The biggest threat to true elimination may be the definition of elimination itself. It tempts authorities to underreport or misclassify cases as the elimination threshold is reached for a given reporting unit. Such a situation may arise in EA as well and distract from sustaining rigorous surveillance. We lack sufficient understanding of transmission dynamics to be confident VL will remain suppressed for a predictable length of time once incidence is driven below a defined threshold. A simpler approach may be to leave the declaration of EPHP to a realistic independent assessment at predefined intervals, the contours and indicators of which adjust as we accumulate technical and pragmatic wisdom over time—or defined in broader terms to include other morbidity measures such as DALYs, along with measures of how effectively systemic efforts have mitigated human suffering.

**Box 1. Recommendations for East African VL elimination programs**

**Table:** 

***Surveillance and case detection*** • Baseline assessments of incidence and epidemiological patterns, care-seeking, and vector ecology, accounting for the considerable heterogeneity, will be invaluable and should inform decentralized elimination strategies. Nimble monitoring systems should be developed based on these assessments to track critical processes and enable rational tweaks to operational plans. • Early establishment of a hybrid—manual and digital—case listing and tracking system that eliminates duplication in reporting while ensuring that all individual cases remain traceable, will provide confidence in case numbers and distribution. • Given the clustered nature of the disease, responsive active case detection strategies targeting recently affected communities, starting with index cases and using snowballing and relevant local information sources, are likely to be effective in reducing time to diagnosis. • Waning of the sensitivity of surveillance with reducing incidence must be anticipated. Embedding robust quality-of-surveillance indicators at the earliest (such as proportion of prolonged fevers screened for VL and proportion of outpatients tested for VL) will mitigate the loss. • It is easy to lose track of suspect and uncertain cases, especially in a context of high population movement; lightly trained community-based surveillance volunteers can be invaluable to sustain tracking. • Patient-centered integrated fever surveillance is an idea mostly untested in large-scale public health programs. EA could be fertile ground for attempting to establish such systems.
***Accurate diagnosis, effective treatment, and shortening the time from onset to treatment*** • Ensuring easy, free-of-cost access to reliable diagnostics and treatment to all vulnerable populations should be considered a cornerstone of the VL elimination strategy in EA. • Investment to develop and deploy rapid diagnostic tests with high sensitivity across EA would greatly increase the likelihood of program success. • Short, highly effective drug regimens need to be developed to decrease the burden on health care systems, ensure full compliance, and decrease likelihood of drug resistance.
***Vector control*** • Deployment of vector control in EA should be based on deeper understanding of vector ecology, distribution, competence, behavior, habitat prevalence, and transmission hotspots. • Proposed integrated vector control strategies that target both peridomestic and sylvatic transmission should be evaluated across major VL-endemic ecological settings and the resulting knowledge shared across the region.
***Confronting case clusters*** • Defining VL ‘outbreaks’ rationally is not simple; calling them case clusters acknowledges their presence without inducing panic. • Given the long and variable incubation periods, and insidious onset of symptoms, available tools for diagnosis, treatment, and vector control are insufficient to immediately end the emergence of new cases. Program leadership should acknowledge and clearly communicate this to avoid ineffective “panic” responses. • Investigations of case clusters can be invaluable in providing insights into local transmission dynamics and for testing intervention strategies. Being ready to initiate investigations quickly will maximize their utility.
***Program governance and responsiveness*** • The elimination program will do well to acknowledge the imperfect knowledge base on which it is attempting the audacious. This will encourage new ideas and experiments around core strategies. • Implementation research combining epidemiology, vector biology, and serology early in the program, preferably in diverse contexts, should lead to rational and cost-effective strategies for elimination. • Program success will be bolstered if multiple, independently funded governmental and nongovernmental partners are involved. • Flexible budgeting in public/government programs is key to enabling availability of adequate resources for decentralized/ contextual strategies. • Uniting partners toward the eventual goal will be critical; attempting one-size-fits-all national plans is likely to prolong time to success.
